# Ionic-Liquid-Based Aqueous Two-Phase Systems Induced by Intra- and Intermolecular Hydrogen Bonds

**DOI:** 10.3390/molecules27165307

**Published:** 2022-08-19

**Authors:** Wenzhuo Xu, Xinpei Gao, Liqiang Zheng, Fei Lu

**Affiliations:** 1 Key Laboratory of Colloid and Interface Chemistry, Shandong University, Ministry of Education, Jinan 250100, China; 2 Key Laboratory of Ministry of Education for Advanced Materials in Tropical Island Resources, Hainan University, No 58 Renmin Avenue, Haikou 570228, China

**Keywords:** ionic liquid, aqueous two-phase system, dicarboxylic acid, amino acid isolation

## Abstract

In recent years, aqueous two-phase systems (ATPSs) have been widely used in different fields and have become an increasingly attractive subject due to their application in the separation and purification of biomolecules. In this work, the aqueous phase behavior of ionic liquids (ILs) was modulated by changing the *cis*-*trans* structure of the anion in ILs. With the same tetra-butyl-phosphine as the cation, the cis-anion exhibited upper critical solution temperature (UCST) phenomena. In contrast, the *trans*-anion exhibited lower critical solution temperature (LCST) phenomena. The proposed mechanism shows that the main factors responsible for these phenomena include variations in the dissociation degree with temperature and the steric hindrance of the ILs. This phase behavior combines the chemical equilibrium in a solution with the microstructure of the molecule and is useful for constructing new chemical dynamic equilibria in ATPS. As an example of its application, aqueous solutions of both ILs can be used for the efficient separation and extraction of specific amino acids. The two ATPS systems reported in this work highlight a simple, effective, and environmentally friendly method for separating small biological molecules.

## 1. Introduction

Ionic liquids (ILs) [1] are a novel class of solvents that possess unique properties such as nonvolatility [2], low toxicity [3,4], ease of handling, nonflammability, and high ionic conductivity. Recently, ILs have received much attention as green media for various chemistry processes [5,6,7]. Over 200 room temperature ILs are known, but the physico-chemical [8,9] data for most ILs are incomplete or lacking. The structure and properties of ILs can be regulated by mixing them with molecular solvents. Such mixtures can regulate systems with good physicochemical properties, and there are complex ionic and intermolecular forces between ILs and molecular solvents. The protonic and non-protonic properties of different compounds in the mixture can also determine the macroscopic state of the system. Among all molecular solvents, water can be a very worthwhile solvent to study because of its unique hydrogen-bonding network. The addition of water to an IL can significantly change its physicochemical properties.

Aqueous two-phase system (ATPS) [10,11,12] is a liquid–liquid fractionation technique and has gained interest because of the great potential for the extraction [13], separation [14,15,16,17], purification [18,19], and enrichment of proteins [20,21,22,23,24,25], membranes, viruses, enzymes [26,27,28,29,30,31], nucleic acids, and other biomolecules both in industry and academia. The upper and lower phases of ATPS are composed of water, in which there are remaining chemicals that cause the upper and lower phases to be in a relative equilibrium. Although the aqueous nature of ATPSs leads to their “greener” properties, conventional polymer-based systems suffer from various problems such as high viscosity and low phase separation rates [32]. In the past two decades, various novel ATPSs have been reported to overcome drawbacks associated with the most conventional systems. In this context, the designable and customizable properties of ILs led to improvements in the phase separation capacity and extraction efficiency of IL-based ATPSs [33,34]. Generally, nearly complete phase separation usually endows IL-based ATPSs with an enhanced, easier recovery and better reusability [35]. Therefore, the phase behavior of IL-based ATPSs has attracted increasing interest in recent years.

The IL-based aqueous systems usually have stimulus-driven dynamic phase transitions, the most common of which is the temperature-driven phase transition. It can be further subdivided into two types: upper critical solution temperature (UCST)-type and lower critical solution temperature (LCST)-type [36,37]. The UCST-type phase transition is a common phenomenon in which the miscibility of two liquids tends to increase with increasing temperature. LCST-type phase transitions are only commonly observed in mixtures with significant differences in molecular sizes, such as the mixtures of polymers and water [38]. Therefore, the property of controlling the phase transition temperature is important in the study of IL-based ATPS, by which the phase transition can be slowly controlled and the system's potential can be extended.

**Figure 1:**
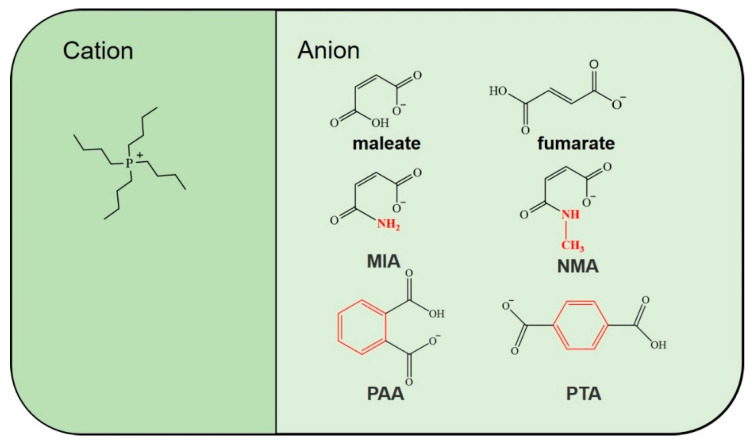
The chemical structures of the examined ILs.

In this work, a class of novel ATPSs has been prepared by a tetra-*n*-butyl phosphonium-type IL and water (Figure 1). Notably, the phase behaviors of these ATPSs are affected by the structure of the anions. The IL [P_4444_][fumarate] with *trans*-anions exhibits an UCST-type phase separation in water, whereas [P_4444_][maleate] with *cis*-anions exhibits a LCST-type behavior in water. The mechanism for the temperature-dependent liquid−liquid phase transition of ATPSs is related to the degree of anion dissociation and the steric hindrance of ILs. Moreover, these ATPSs allow the effective separation of amino acids with specific structures because of their reversible conversion between homogeneous solutions and liquid–liquid partitioned phases.

## 2. Results and Discussions

**Figure 2:**
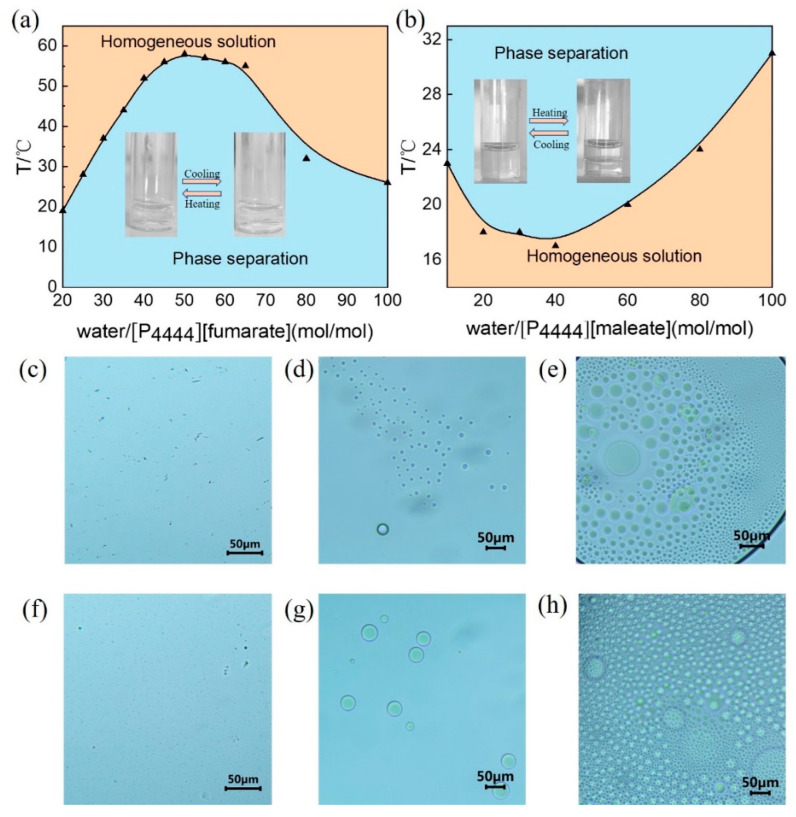
Phase diagrams for two different types of aqueous two-phase systems (**a**) [P_4444_][fumarate] and (**b**) [P_4444_][maleate]. OM pictures of [P_4444_][fumarate]-water system in (**c**) homogeneous solution, (**d**) upper phase and (**e**) lower phase, [P_4444_][maleate]-water system in (**f**) homogeneous solution, (**g**) upper phase and (**h**) lower phase.

The ATPSs of [P_4444_][fumarate] and [P_4444_][maleate] were prepared by mixing the ILs and water. A previous study of the IL [P_4444_][fumarate] revealed that the aqueous solutions of this IL pass from opaque to clear upon heating (UCST-type phase transition), and the phase-separation temperature (T_ps_) depends on the IL concentration [39]. As shown in Figure 2a, the aqueous solution of [P_4444_][fumarate] shows a convex curve giving a UCST type of 58 °C at a water content of 50 (mol/mol), where at the highest temperature, the IL-water system forms a homogeneous solution. In contrast, [P_4444_][maleate] is completely miscible with water at low temperatures and a clear homogeneous solution is observed. The temperature at which the homogeneous solution turns immiscible is the cloud point (T_cp_). As shown in Figure 2b, a typical phase diagram of LCST-type transition is reflected with a concave curve between temperature and mole fraction. The lowest T_cp_ is 18 °C for water content of 40 (mol/mol). Upon cooling the immiscible [P_4444_][maleate]-water system, the volume fraction of the IL phase increases gradually and reaches a homogeneous phase, suggesting an increase in solubility of the IL by cooling.

From a thermodynamic point of view, these IL-water systems spontaneously tend to minimize their free energy by minimizing energy and maximizing entropy [40]. For the UCST-type phase transition, the value of free energy change (ΔG_m_) is zero when the ATPS reaches the T_ps_, at which T_ps_ can be expressed as the ratio of ΔH_m_ to ΔS_m_. When the solution temperature is low, the contribution of the entropic effect is minimal. As the concentration of IL increases, the intermolecular hydrogen bonding force between IL and water increases, leading to an increase in enthalpic interaction. When T_ps_ reaches its highest point, the increase in IL content strengthens the electrostatic interaction between ILs, and its effect on entropy makes T_ps_ more inclined to decrease. This is a reason why the convex curve is displayed in the UCST-type. In contrast, LCST-type phase transitions involve strong interactions between individual components. When the temperature is low enough, the loss of ΔH_m_ plays a dominant role on the minimum point in T_cp_, showing a concave curve in the phase diagram.

The optical microscope (OM) can provide a direct observation of the aggregates in different phases. The morphologies of aggregates for [P_4444_][fumarate] and [P_4444_][maleate] in water were examined with OM. When the temperature is controlled at the homogeneous phase for both [P_4444_][fumarate] and [P_4444_][maleate], no aggregate can be observed, as shown in Figure 2c,f, respectively. Figure 2d,g provide the representative images in the upper phase of the [P_4444_][fumarate]-water and [P_4444_][maleate]–water systems, respectively, which clearly show spherical structures. Moreover, in the IL-rich area of the lower phase, the morphology of the aggregates remains spherical, and a tendency of small droplets to aggregate is observed (Figure 2e,h).

To verify the main driving forces for the formation of ATPSs, a series of ILs with similar structures was designed and synthesized by acid–base neutralization reaction (Figure 1 and Figures S1–S4 from Supplementary Materials). It is worthy to point out that [P_4444_][PTA] and [P_4444_][PAA] with benzene rings cannot form homogeneous phases with water due to their strong hydrophobicity. However, [P_4444_][MIA] and [P_4444_][NMA] have more hydrophilic anions and are in a homogeneous state without any phase separation. This clearly demonstrates that the hydrophobicity of the ILs determines the formation of ATPSs firstly.

**Figure 3:**
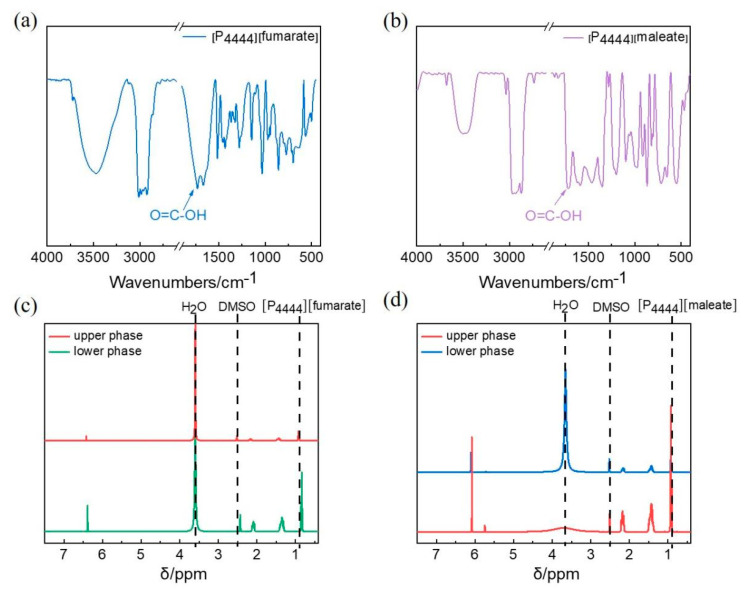
The IR spectra of (**a**) [P_4444_][fumarate] and (**b**) [P_4444_][maleate]. The ^1^H-NMR spectra of the upper and lower phases of ATPS (**c**) [P_4444_][fumarate] and (**d**) [P_4444_][maleate].

In order to further explore the effect of cation–anion interactions on the phase behavior of ATPSs, the FTIR spectra of [P_4444_][fumarate] and [P_4444_][maleate] were carried out. It can be seen from Figure 3a,b that the peak at 1710 cm^−1^ attributed to the carboxylic acid group in IL anion appears for both [P_4444_][fumarate] and [P_4444_][maleate] samples [40]. It indicates that the tetrabutylphosphine cations can bind to acidic anions in equimolar proportions, and a carboxylic acid group is exposed accordingly. A range of distributional equilibria may exist in the solution, so it is important to explore the composition of the two phases. To further verify the components of the upper and lower phases of ATPS, the samples from the two phases were examined using ^1^H-NMR spectra at 25 °C (Figure 3c,d). From the magnitude of the different peaks in the spectra, it can be seen that the phase separation is associative, for both [P_4444_][fumarate] and [P_4444_][maleate], in which the upper phase is rich-water and the lower phase is rich-IL. Interestingly, the proportion of water in the lower phase of [P_4444_][fumarate] reaches 7.05% and is higher than that in the lower phase of [P_4444_][maleate]. This phenomenon is attributed to the trans configuration of the anion in [P_4444_][fumarate], which makes it easier for small molecules of water to bind.

**Figure 4:**
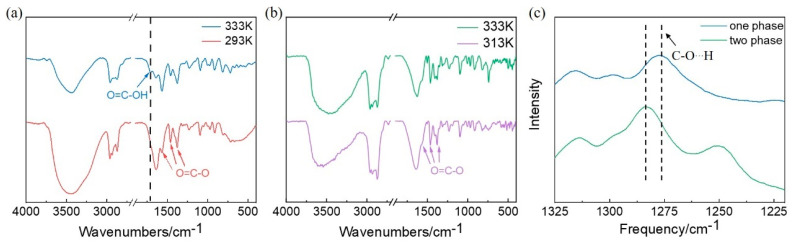
(**a**) IR spectra of [P_4444_][fumarate] at 293 K (red) and 333 K (blue). (**b**) IR spectra of [P_4444_][PTA] at 313 K (purple) and 333 K (green). (**c**) Raman spectra for aqueous solutions of [P_4444_][fumarate] before and after phase separation. When the IL-water system is phased, the IL-rich phase was selected for analysis.

To account for the different phase transitions in aqueous solutions of [P_4444_][fumarate] and [P_4444_][maleate], the interaction between IL and water was further explored. The IR spectra of [P_4444_][fumarate] were measured at different temperatures in Figure 4a. In the IR spectra of [P_4444_][fumarate], the bands at 1354 cm^−1^, 1460 cm^−1,^ and 1566 cm^−1^ are attributed to the O=C-O ring stretching vibration [41]. In the IR spectrum of one-phase [P_4444_][fumarate] solution at 333 K, the peak at 1711 cm^−1^ may be attributed to the O=C stretching vibration band of OH. However, when the temperature decreases to 293 K, the peak at 1711 cm^−1^ disappears, indicating the low dissociation of [P_4444_][fumarate] in an aqueous two-phase solution. Based on the IR data, the internal microstructures of IL–water networks are proposed. Upon decreasing the temperature, the dissociation of [P_4444_][fumarate] likely inhibits the binding of [P_4444_][fumarate] and water. The higher dissociation degree of IL generates a stronger electrostatic interaction between anions and cations, which induces the formation of IL clusters [42]. The proposed mechanism is also in accordance with our OM results that some aggregates can be observed in the ATPS of [P_4444_][fumarate]. Furthermore, the increasing temperature would induce a gradual decrease in the dissociation of fumarate anion, resulting in an enhanced intermolecular hydrogen bonding between [P_4444_][fumarate] and water. From then on, the IL-water interactions are progressively stronger than IL-IL interactions, and finally, the enhancement of IL solubility leads to the generation of homogeneous aqueous solutions.

To examine the influence of intermolecular hydrogen bonds on the formation of ATPS, [P_4444_][PTA], which has a similar structure to [P_4444_][fumarate], was chosen to explore its phase behavior. Its benzene ring structure allows it to have a larger π-π conjugation system, and a carboxyl substituent at the opposite position in the benzene ring can also provide suitable conditions for the formation of intermolecular hydrogen bonds. The IR spectra of [P_4444_][PTA]-water system were collected and shown in Figure 4b. Similarly to the [P_4444_][fumarate] aqueous solution, the peaks at 1340 cm^−1^, 1457 cm^−1^, and 1577 cm^−1^ of O=C-O stretching vibration were observed. Combined with the IR spectra of [P_4444_][fumarate]-water system, it can be determined that the [P_4444_][PTA] also has a large degree of dissociation in water. The tethering of cation and anion results in very strong intermolecular interaction and leads to phase separation.

Raman spectroscopy is a powerful tool to probe the interaction and association of molecules. As seen in Figure 4c, the Raman spectra of [P_4444_][fumarate] in different phase has been studied. Considering the dissociation of IL is closely related to the condition of the C-O bond, the spectral region of 1200–1400 cm^−1^ that contains the typical peaks of C-O…H at 1270 cm^−1^ was monitored. For the homogeneous phase of [P_4444_][fumarate], the low dissociation degree of IL allows the peak of C-O…H to be observed in the Raman spectrum. When the solution changes from homogeneous to two-phase, an obvious blue shift for C-O…H can be observed due to the high density of electron cloud generated by the increased dissociation of fumaric acid.

**Figure 5:**
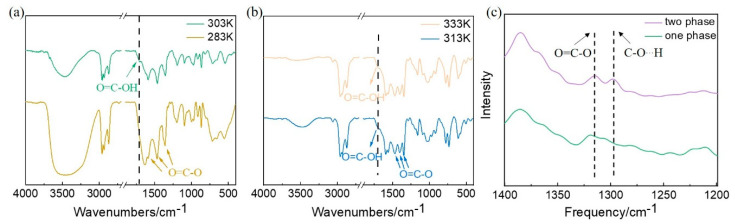
(**a**) The IR spectra of [P_4444_][maleate] at 283 K (yellow) and 303 K (green). (**b**) The IR spectra of [P_4444_][PAA] at 313 K (blue) and 333 K (yellow). (**c**) The Raman spectra for aqueous solutions of [P_4444_][maleate] before and after phase separation. When the IL-water system is phased, the IL-rich phase was selected for analysis.

To understand the effect of the *cis*-*trans* structure of IL anion on the phase transition of ATPS, the IR spectra of the [P_4444_][maleate]-water system before and after the phase separation were also examined and shown in Figure 5a. The peak at 1350 cm^−1^, 1476 cm^−1^, and 1587 cm^−1^ can be assigned to O=C-O ring stretching vibration, and the peak at 1714 cm^−1^ can be assigned to the stretching vibration of C=O in the carboxyl group of maleate anions. Similarly to fumaric anions, the degree of dissociation of maleate anions is also temperature-dependent. The disappearance of the peak of O=C…OH at 283 K demonstrates the substantial dissociation of dicarboxylic acid at this temperature. Considering the steric hindrance of *cis*-maleate anions, the interactions of IL-IL tend to be replaced by the hydrogen bonding interactions between anions and water. Thus, IL molecules are less prone to forming self-assemblies, and water molecules are isolated from each other as they are involved in hydrogen bonding with COO^-^. This situation corresponds to the homogeneous phase in ATPS. When the temperature rises above T_cp_, the low dissociation of maleate anions could generate a possible intramolecular hydrogen bond between the undissociated -COOH group. In contrast to the intermolecular hydrogen bonding between IL and water, the intramolecular hydrogen bonding of the IL anion becomes prominent. Hence, [P_4444_][maleate] molecules prefer to self-assembly into clusters, which become excluded from water and form the two phases at the macro-level.

To gain a deeper insight into the role of intramolecular hydrogen bonds on the phase separation in ATPS, the IR spectra of IL [P_4444_][PAA] with similar *cis*-anions were also explored. As shown in Figure 5b, the characteristic peaks of O=C-O can be found in both spectra at 313 K and 333 K. In addition, the peak at 1703 cm^−1^ corresponding to the O=C stretching band of OH can also be observed in both spectra, indicating the almost identical dissociation degree of carboxyl groups at both temperatures. Due to the intramolecular hydrogen bonding between the permanent -COOH group, [P_4444_][PAA] shows a tendency to form a separated phase with water.

The Raman spectra of [P_4444_][maleate] were further conducted to investigate the dissociation of the acidic group in maleate anions. As shown in Figure 5c, the characteristic peak at 1310 cm^−1^ ascribed to O=C-O can be observed in both one-phase solution and the lower phase of ATPS. While the peak at 1295 cm^−1^ ascribed to C-O…H can only be found in the lower phase of ATPS, indicating the lesser dissociation of carboxylic acid in maleate anions. Furthermore, the reduction in the electron cloud density due to the low dissociation degree also generates a red shift of the peak for O=C-O. This result is in accordance with the above IR analysis that the dissociation of the acidic group decreases with increasing temperatures. Taking the steric configuration of *cis*-anions into consideration, the formation of intramolecular hydrogen bonding between anions is favorable and further results in the aggregation of ILs.

**Figure 6:**
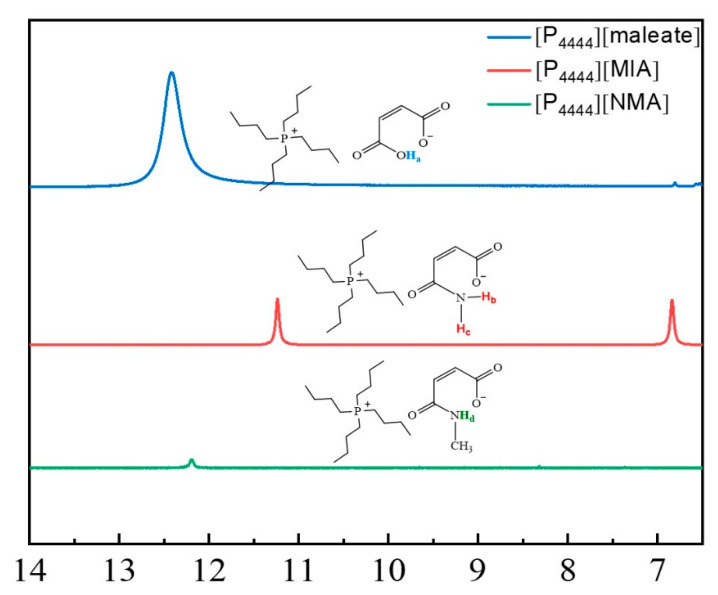
^1^H-NMR results of different ILs with possible intramolecular hydrogen bonding interactions.

^1^H-NMR spectra provide more detailed information about the chemical environment of the H atom and, thus, provide an insight into the hydrogen bonding interactions of molecules. Increasing the strength of the hydrogen bonding interactions can induce chemical shifts toward downfield because the protons are more strongly deshielded when stronger hydrogen bonds participate [43]. Figure 6 shows the ^1^H-NMR spectra of [P_4444_][maleate], and two ILs with similar *cis*-anionic structures, [P_4444_][MIA] and [P_4444_][NMA]. To examine the presence of hydrogen bonds in [P_4444_][maleate], ^1^H-NMR spectra were recorded with D_2_O as an external chemical shift reference in the capillaries tube. The H_a_ of [P_4444_][maleate] showed a distinct peak at 12.51 ppm on the spectrum. Compared to the O-H without hydrogen bonding at 11.00 ppm, the downfield chemical shift proves the existence of hydrogen bonding [44]. In general, non-protic solvents such as DMSO-d_6_ will show better defined N-H signals than protic D_2_O for [P_4444_][MIA] and [P_4444_][NMA]. For [P_4444_][MIA], H_b_ and H_c_ exhibit signals at 11.23 and 6.85 ppm, respectively, demonstrating that the protons attached to N are in different chemical environments, in which H_b_ may become involved in the formation of intramolecular hydrogen bonds. Similarly, the H_d_ of [P_4444_][NMA] at 12.54 ppm also indicates the existence of intramolecular hydrogen bonding in the NH group.

**Figure 7:**
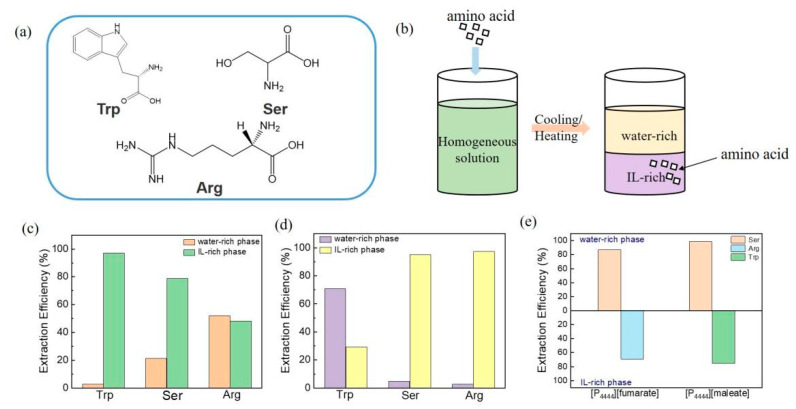
(**a**) Chemical structures of the used amino acids. (**b**) Schematic diagram of amino acid extraction. Extraction efficiency of IL on amino acid molecules in (**c**) [P_4444_][fumarate]-water system and (**d**) [P_4444_][maleate]-water system. (**e**) Distribution of amino acids in ATPS.

Because of the excellent phase separation ability of [P_4444_][fumarate] and [P_4444_][maleate] based ATPSs, the extraction performances for bioactive amino acids were further evaluated. Tryptophan (Trp), serine (Ser), and arginine (Arg) are essential amino acids that are widely used in food and biochemical research fields [45]. The chemical structures of the amino acids used are represented in Figure 7a. As shown in Figure 7b, amino acids were added to [P_4444_][fumarate]-based and [P_4444_][maleate]-based ATPS system with a water/IL molar ratio of 40 at 333 K and 283 K, respectively. After thorough stirring, the temperature was adjusted to 298 K to produce phase separation to concentrate amino acids in one phase to achieve extraction. The extraction results of the two ILs are shown in Figure 7c,d. These data are shown in percentage extraction efficiencies (EE%) of each amino acid for each phase, and EE% represents the equivalent of the percentage ratio between the amount of each amino acid in a phase and in the total mixture (Tables S1–S3). For the [P_4444_][fumarate]-water system, Trp and Ser are favorable for concentration in the IL-rich phase, with extraction efficiencies of 97.19% and 78.68%, respectively. We proposed that the hydrophobic interactions between the aromatic group of Trp and [P_4444_][fumarate] are the main forces. However, in the [P_4444_][maleate]-based ATPS, Trp prefers to concentrate in the water-rich phase instead of the IL-rich phase(EE% = 70.79%), while Ser is still favorable to concentrate in the IL-rich phase with an extraction efficiency of 97.30%.

Furthermore, the ATPSs were used to separate the mixture of two amino acids. Briefly, two amino acids were added to the homogenous IL-water system and stirred. Then, the phase splitting was attained by changing the temperature and the amino acid would be dissolved in a different phase to realize the separation. As shown in Figure 7e, the extraction efficiencies of the [P_4444_][fumarate]-based ATPS for the separation of Ser and Arg are 86.73% and 69.23%, respectively, demonstrating that the two amino acids can be efficiently separated by the IL-water system after mixing. In addition, the [P_4444_][maleate]-based ATPS results in very high extraction efficiencies with the value of 98.88% and 75% for Ser and Trp, which means Ser could be almost separated in a one-stage extraction process.

## 3. Materials and Methods

### 3.1. Materials

Tetra-*n*-butyl phosphonium hydroxide ([P_4444_] [OH]) (40 wt% in water), phthalate (PAA) (99%), and amino acids used in the experiment were purchased from Aladdin (Shanghai, China). Fumarate (99%) and maleate (99%) were purchased from J&K. Terephthalic acid (PTA) (99%) was purchased from TCI. Maleamidic acid (MIA) (99%) was purchased from Aladdin. *N*-Maleamidic acid (NMA) (99%) was purchased from Aladdin. D_2_O (99%) was purchased from Macklin (Shanghai, China). Aqueous solutions were prepared using water of 18 MΩ × cm.

### 3.2. Synthesis of the Ionic Liquid (IL)

For [P_4444_][fumarate], fumaric acid was first dissolved in ultrapure water, then an equimolar amount of tetra butyl phosphine hydroxide was dissolved in water and added dropwise to the above solution, and the mixture was stirred at room temperature for 24 h. After evaporation to remove the water, the product was recrystallized three times in ethyl acetate–ethanol solution and dried under vacuum at room temperature for 48 h. In contrast, [P_4444_][maleate], [P_4444_][MIA], [P_4444_][NMA], [P_4444_][PTA], and [P_4444_][PAA] were all reacted by first dissolving the anionic acid in water, adding an equimolar amount of tetra butyl phosphine hydroxide and stirring for 24 h at room temperature. The product was obtained by dichloromethane extraction in an aqueous solution, the dichloromethane was removed by spin evaporation, and the product was dried under vacuum at room temperature for 48 h. The structure of the obtained ionic liquid was determined by ^1^H NMR spectroscopy (400 Hz, D_2_O).

[P_4444_][fumarate] (relative to TMS): 0.77 (12H, t, CH_2_CH_3_), 1.27–1.40 (16H, m, CH_2_CH_3_), 2.01 (8H, m, CH_2_CH_2_), 6.58 (2H, s, CH=CH).

[P_4444_][maleate] (relative to TMS): 0.74 (12H, t, CH_2_CH_3_), 1.24–1.37 (16H, m, CH_2_CH_3_), 2.00 (8H, m, CH_2_CH_2_), 6.16 (2H, s, CH=CH).

[P_4444_][MIA] (relative to TMS): 0.93 (12H, t, CH_2_CH_3_), 1.39–1.47 (16H, m, CH_2_CH_3_), 2.19 (8H, m, CH_2_CH_2_), 5.35 (1H, s, CH=CH), 6.05 (1H, s, CH=CH), 6.83 (1H, s, N-H), 12.51 (1H, s, N-H).

[P_4444_][NMA] (relative to TMS): 0.78 (12H, t, CH_2_CH_3_), 1.27–1.42 (16H, m, CH_2_CH_3_), 2.00 (8H, m, CH_2_CH_2_), 2.61 (3H, t, CH_3_), 5.79 (1H, s, CH=CH), 6.16 (3H, s, CH=CH), 12.54 (1H, s, N-H).

[P_4444_][PAA] (relative to TMS): 0.89 (12H, t, CH_2_CH_3_), 1.35–1.49 (16H, m, CH_2_CH_3_), 2.15–2.23 (8H, m, CH_2_CH_2_), 7.49–7.52 (2H, t, Ar-H), 8.19–8.21 (2H, t, Ar-H).

[P_4444_][PTA] (relative to TMS): 0.93 (12H, t, CH_2_CH_3_), 1.37–1.48 (16H, m, CH_2_CH_3_), 2.14–2.21 (8H, m, CH_2_CH_2_), 7.80 (4H, d, Ar-H).

### 3.3. Characterization

The critical dissolution temperature was determined by constant heating in a ZNCL-G190 × 90 water bath apparatus. ^1^H-NMR spectra were recorded at 400 MHz using a Bruker Advance 400 spectrometer, and for [P_4444_][maleate], the NMR hydrogen spectrum of H in the hydrogen bond was determined by adding a capillary filled with D_2_O to the NMR tube containing the IL sample. Fourier transform infrared (FT-IR) spectra were obtained using a Bruker Tensor II spectrometer, and spectral data at different temperatures were determined using a variable temperature accessory. Raman spectral data were obtained using a LabRAM HR Evolution from HORIBA, and for liquid samples, they were charged in capillary tubes of 0.9 mm diameter before testing. The extraction efficiency of amino acids was calculated by measuring the integral ratio of amino acids to IL in the ^1^H-NMR spectra of the upper and lower phases of ATPS before and after dissolution.

## 4. Conclusions

In summary, we have prepared a series of ILs with *cis*/*trans* dicarboxylic acid anions and investigated the ATPS phase transition mechanism. It has been found that the conformational changes of IL anions endow [P_4444_][fumarate] with UCST behavior while [P_4444_][maleate] is endowed with LCST behavior in aqueous solutions. The electrostatic interactions between cations and anions of ILs compete with the interactions between IL and water, leading to different critical dissolution temperatures. When the anion–cation interaction is greater than the IL-water interaction, IL molecules are favorable for aggregation to form a separated liquid–liquid phase. Otherwise, the IL-water system would form a homogeneous solution. In addition, the dissociation of the dicarboxylic acid in anions and the spatial resistance in the presence of hydrogen bonds have an impact on the phase-separation behavior of ATPS. Finally, the reversible transformation of ATPS between homogeneous and two phases provides an effective platform for the separation of small amino acid molecules. This work is expected to provide some theoretical guidelines for the phase behavior of ILs.

## Data Availability

Not applicable.

## Supplementary Materials

The following supporting information can be downloaded at: www.mdpi.com/article/10.3390/molecules27165307/s1, Figure S1: ^1^H-NMR spectrum of [P_4444_][MIA], Figure S2: ^1^H-NMR spectrum of [P_4444_][NMA], Figure S3: ^1^H-NMR spectrum of [P_4444_][PAA], Figure S4: ^1^H-NMR spectrum of [P_4444_][PTA], Table S1: [P_4444_][fumarate] extracts the relative amounts of amino acids, IL and water used in the experiment (prepare ATPS system with 0.001 mol IL and 0.04 mol water, add 0.0001 mol amino acid), Table S2: [P_4444_][maleate] extracts the relative amounts of amino acids, IL and water used in the experiment (prepare ATPS system with 0.001 mol IL and 0.04 mol water, add 0.0001 mol amino acid), Table S3: The relative amounts of amino acids, IL and water used in the extraction of mixed amino acids by [_P4444_][fumarate] and [P_4444_][maleate] (prepare ATPS system with 0.001 mol IL and 0.04 mol water, add 0.0001 mol amino acid).

## Sample Availability

Samples of the compounds are not available from the authors.
