# Supplementary material for: Ionic-Liquid-Based Aqueous Two-Phase Systems Induced by Intra- and Intermolecular Hydrogen Bonds
Source: Molecules. 2022 Aug 19;27(16):5307. doi: 10.3390/molecules27165307 (PMC9414173; doi:10.3390/molecules27165307)
Supplement: Supplementary file 1 [file molecules-27-05307-s001.zip › molecules-1865167-supplementary materials.pdf]

SUPPLEMENTARY MATERIAL

Ionic-Liquid-Based Aqueous Two-Phase Systems Induced by Intra- and Intermolecular Hydrogen Bonds

Wenzhuo Xu [1], Xinpei Gao [2], Liqiang Zheng [1,] and Fei Lu [2,*]*

[1]Key Laboratory of Colloid and Interface Chemistry, Shandong University, Ministry of Education, Jinan, 250100, P. R. China.

[2]Key Laboratory of Ministry of Education for Advanced Materials in Tropical Island Resources, Hainan University, No 58, Renmin Avenue, Haikou 570228, P. R. China.

Corresponding Author

*Fei Lu; E-mail address: lufei@hainanu.edu.cn

*Liqiang Zheng; E-mail address: lqzheng@sdu.edu.cn

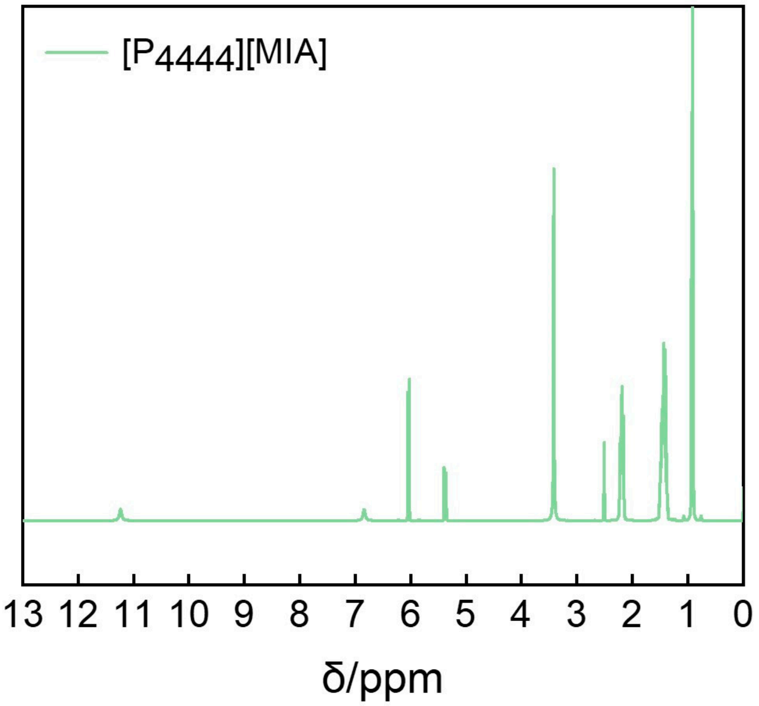

Figure S1. ^{1}H NMR spectrum of [P_{4444}][MIA].

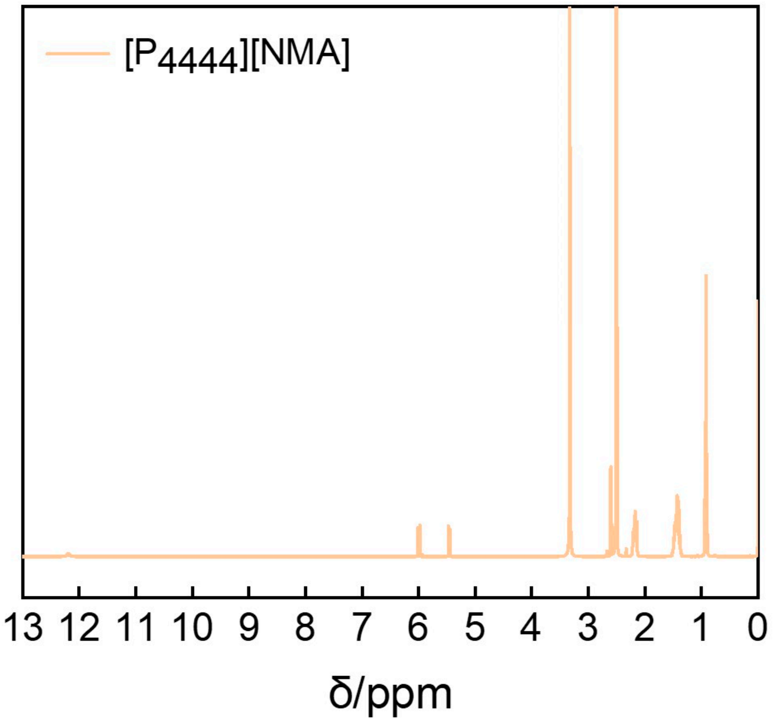

Figure S2. ^{1}H NMR spectrum of [P_{4444}][NMA].

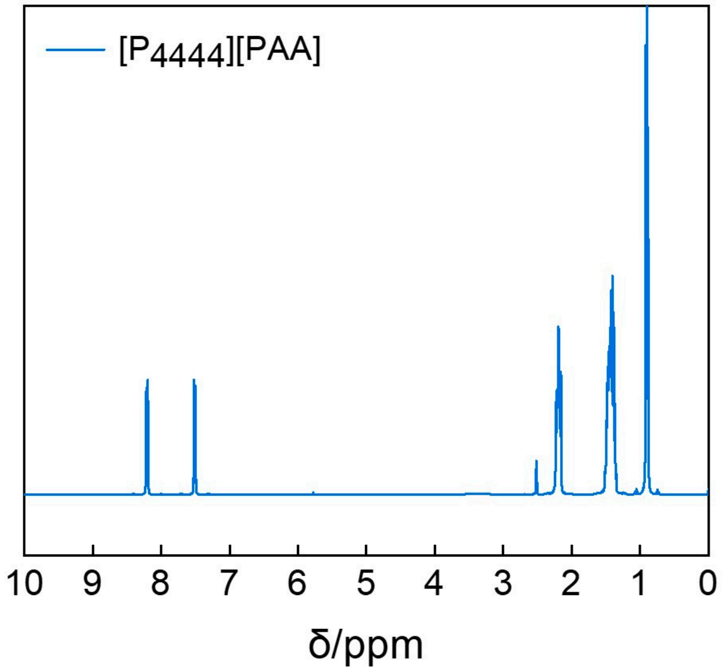

Figure S3. ^{1}H NMR spectrum of [P_{4444}][PAA].

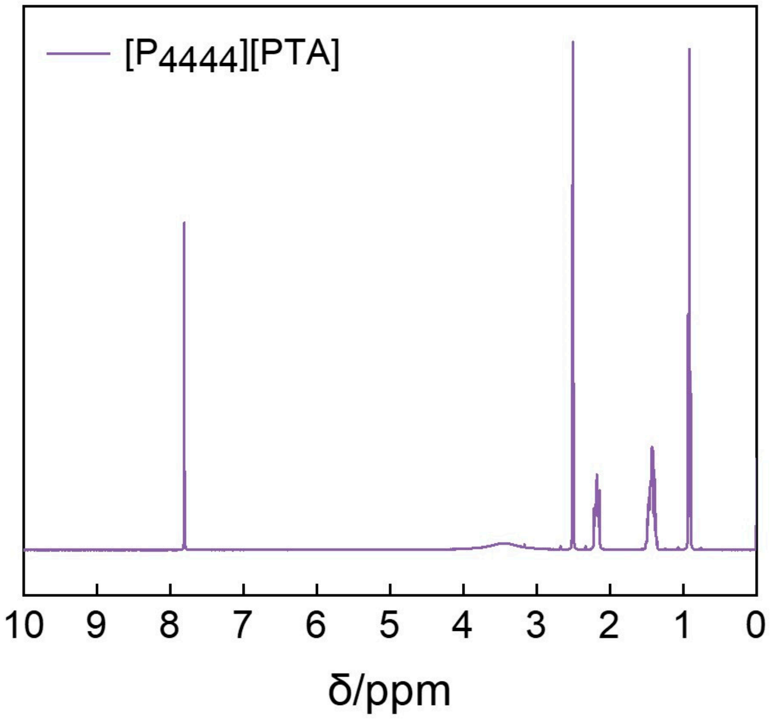

Figure S4. ^{1}H NMR spectrum of [P_{4444}][PTA].

Table S1. [P_{4444}][fumarate] extracts the relative amounts of amino acids, IL and water used in the experiment (prepare ATPS system with 0.001mol IL and 0.04mol water, add 0.0001mol amino acid).

Types of amino acids	The water-rich phase			The IL-rich phase		
	The amount of amino acid/ mol	The amount of IL/ mol	The amount of water/ mol	The amount of amino acid/ mol	The amount of IL/ mol	The amount of water/ mol
Trp	$2.81*10^{-6}$	$7.05*10^{-5}$	$3.27*10^{-2}$	$9.72*10^{-5}$	$9.29*10^{-4}$	$7.31*10^{-3}$
Ser	$2.13*10^{-5}$	$5.31*10^{-5}$	$3.06*10^{-2}$	$7.87*10^{-5}$	$9.47*10^{-4}$	$9.45*10^{-3}$
Arg	$5.18*10^{-5}$	$1.24*10^{-4}$	$2.78*10^{-2}$	$4.81*10^{-5}$	$8.76*10^{-4}$	$1.22*10^{-2}$

Table S2. [P_{4444}][maleate] extracts the relative amounts of amino acids, IL and water used in the experiment (prepare ATPS system with 0.001mol IL and 0.04mol water, add 0.0001mol amino acid).

Types of amino acids	The water-rich phase			The IL-rich phase		
	The amount of amino acid/ mol	The amount of IL/ mol	The amount of water/ mol	The amount of amino acid/ mol	The amount of IL/ mol	The amount of water/ mol
Trp	$7.08*10^{-5}$	$1.08*10^{-4}$	$3.04*10^{-2}$	$2.92*10^{-5}$	$8.92*10^{-4}$	$9.67*10^{-3}$
Ser	$4.84*10^{-6}$	$4.76*10^{-5}$	$3.76*10^{-2}$	$9.52*10^{-5}$	$9.52*10^{-4}$	$2.43*10^{-3}$
Arg	$2.70*10^{-6}$	$8.63*10^{-5}$	$3.51*10^{-2}$	$9.73*10^{-5}$	$9.14*10^{-4}$	$4.90*10^{-3}$

Table S3. The relative amounts of amino acids, IL and water used in the extraction of mixed amino acids by [P_{4444}][fumarate] and [P_{4444}][maleate] (prepare ATPS system with 0.001mol IL and 0.04mol water, add 0.0001mol amino acid).

Types of ATPS		The water-rich phase			The IL-rich phase		
		The amount of amino acid/ mol	The amount of IL/ mol	The amount of water/ mol	The amount of amino acid/ mol	The amount of IL/ mol	The amount of water/ mol
[P_{4444}][fumarate]-water system	Ser	$8.67*10^{-5}$	$6.35*10^{-5}$	$3.13*10^{-2}$	$1.33*10^{-5}$	$9.36*10^{-4}$	$8.74*10^{-3}$
	Arg	$3.08*10^{-5}$	$1.39*10^{-4}$	$2.73*10^{-2}$	$6.92*10^{-5}$	$8.61*10^{-4}$	$1.27*10^{-2}$
[P_{4444}][maleate]-water system	Ser	$9.89*10^{-5}$	$5.48*10^{-5}$	$3.63*10^{-2}$	$1.13*10^{-6}$	$9.45*10^{-4}$	$3.75*10^{-3}$
	Trp	$2.50*10^{-5}$	$8.51*10^{-5}$	$3.36*10^{-2}$	$7.50*10^{-5}$	$9.14*10^{-4}$	$6.42*10^{-3}$
